# Supplementary material for: Incidence and Characteristics of Kidney Stones in Patients on Ketogenic Diet: A Systematic Review and Meta-Analysis
Source: Diseases. 2021 May 25;9(2):39. doi: 10.3390/diseases9020039 (PMC8161846; doi:10.3390/diseases9020039)
Supplement: Supplementary file 1 [file diseases-09-00039-s001.zip › diseases-1215736-supplementary.pdf]

Database: Ovid MEDLINE(R)
Search Strategy:
----
1 ketogenic diet.mp.
2 exp ketogenic diet/
3 keto diet.mp.
4 atkins diet.mp.
5 exp atkins diet/
6 low carb diet.mp.
7 low carbohydrate diet.mp
8 exp low carbohydrate diet/
9 1 or 2 or 3 or 4 or 5 or 6 or 7 or
10 nephrolithiasis.mp.
11 exp nephrolithiasis/
12 kidney stone.mp.
13 exp kidney stone/
14 kidney stones.mp
15 exp kidney stones/
16 10 or 11 or 12 or 13 or 14 or 15
17 9 and 16

EMBASE and Cochrane Database of Systematic Reviews
SEARCH QUERY
('ketogenic diet' OR 'keto diet' OR 'atkins diet' OR 'low carb diet' OR 'low carbohydrate diet') AND (nephrolithiasis OR 'kidney stone' OR 'kidney stones')

PRISMA 2009 Checklist

Section/topic	#	Checklist item	Reported on page #
**TITLE**			
Title	1	Identify the report as a systematic review, meta-analysis, or both.	Title page
**ABSTRACT**			
Structured summary	2	Provide a structured summary including, as applicable: background; objectives; data sources; study eligibility criteria, participants, and interventions; study appraisal and synthesis methods; results; limitations; conclusions and implications of key findings; systematic review registration number.	2
**INTRODUCTION**			
Rationale	3	Describe the rationale for the review in the context of what is already known.	4
Objectives	4	Provide an explicit statement of questions being addressed with reference to participants, interventions, comparisons, outcomes, and study design (PICOS).	4
**METHODS**			
Protocol and registration	5	Indicate if a review protocol exists, if and where it can be accessed (e.g., Web address), and, if available, provide registration information including registration number.	4
Eligibility criteria	6	Specify study characteristics (e.g., PICOS, length of follow-up) and report characteristics (e.g., years considered, language, publication status) used as criteria for eligibility, giving rationale.	5
Information sources	7	Describe all information sources (e.g., databases with dates of coverage, contact with study authors to identify additional studies) in the search and date last searched.	4-5
Search	8	Present full electronic search strategy for at least one database, including any limits used, such that it could be repeated.	4-5, Item S
Study selection	9	State the process for selecting studies (i.e., screening, eligibility, included in systematic review, and, if applicable, included in the meta-analysis).	4-5, figure 1
Data collection process	10	Describe method of data extraction from reports (e.g., piloted forms, independently, in duplicate) and any processes for obtaining and confirming data from investigators.	4-5
Data items	11	List and define all variables for which data were sought (e.g., PICOS, funding sources) and any assumptions and simplifications made.	4-5
Risk of bias in individual	12	Describe methods used for assessing risk of bias of individual studies (including specification of whether this was	6-7

studies		done at the study or outcome level), and how this information is to be used in any data synthesis.	
Summary measures	13	State the principal summary measures (e.g., risk ratio, difference in means).	6-7, Figure 2-4
Synthesis of results	14	Describe the methods of handling data and combining results of studies, if done, including measures of consistency (e.g., I^2) for each meta-analysis.	6
